# Examination of Fas-Induced Apoptosis of Murine Thymocytes in Thymic Tissue Slices Reveals That Fas Is Dispensable for Negative Selection

**DOI:** 10.3389/fcell.2020.586807

**Published:** 2020-10-21

**Authors:** Chang-Feng Chu, Hsing-Kai Feng, Kuang-Hui Sun, Chia-Lin Hsu, Ivan L. Dzhagalov

**Affiliations:** ^1^Institute of Microbiology and Immunology, National Yang-Ming University, Taipei, Taiwan; ^2^Department of Biotechnology and Laboratory Science in Medicine, National Yang-Ming University, Taipei, Taiwan

**Keywords:** Fas, thymocytes, apoptosis, negative selection, FasL, caspase 3, phosphatidylserine, efferocytosis

## Abstract

The death receptor Fas can induce cell death through the extrinsic pathway of apoptosis in a variety of cells, including developing thymocytes. Although Fas-induced cell death has been researched and modeled extensively, most of the studies have been done *in vitro* because of the lethality of Fas triggering *in vivo*. Thus, little is known about the time line of this type of cell death *in vivo*, specifically, how does the presence of macrophages and pro-survival cytokines affect apoptosis progression. In addition, although the sequence and timing of events during intrinsic pathway activation in thymocytes *in situ* have been described, no corresponding data for the extrinsic pathway are available. To address this gap in our knowledge, we established a novel system to study Fas-induced thymocyte cell death using tissue explants. We found that within 1 h of Fas ligation, caspase 3 was activated, within 2 h phosphatidylserine was externalized to serve as an “eat-me” signal, and at the same time, we observed signs of cell loss, likely due to efferocytosis. Both caspase 3 activation and phosphatidylserine exposure were critical for cell loss. Although Fas ligand (FasL) was delivered simultaneously to all cells, we observed significant variation in the entry into the cell death pathway. This model also allowed us to revisit the role of Fas in negative selection, and we ruled out an essential part for it in the deletion of autoreactive thymocytes. Our work provides a timeline for the apoptosis-associated events following Fas triggering *in situ* and confirms the lack of involvement of Fas in the negative selection of thymocytes.

## Introduction

Apoptosis is a fundamental biological process that is particularly important for the immune system (Nagata, 2018). Two major apoptotic pathways have been identified – the intrinsic and the extrinsic pathway – that converge on the activation of caspase 3, a protease that is central in dismantling the cell. The intrinsic pathway is activated by various stimuli such as growth factor deprivation, genotoxic stress, or developmental cues. The death signal leads to permeabilization of the outer mitochondrial membrane controlled by members of the Bcl-2 family and the release of molecules that lead to the activation of caspase 3 (Singh et al., 2019). The extrinsic pathway is initiated by ligation of death receptors on cell surface such as Fas (Strasser et al., 2009). Upon ligation by FasL, Fas recruits the adapter FADD that interacts with caspase 8. The oligomerization of caspase 8 leads to its activation, and in several types of cells, including lymphocytes, activated caspase 8 directly cleaves and activates caspase 3, a “point of no return” in apoptosis. Caspase 3 has >1000 substrates (Nagata, 2018), and their destruction interrupts vital cell processes and marks the cell for efferocytosis by macrophages through the production of “eat-me” signals. The best-studied “eat-me” signal is the phospholipid phosphatidylserine (PS) that is typically confined to the inner leaflet of the plasma membrane by the action of the flippases ATP11A and ATP11C (Segawa et al., 2014). Upon apoptosis induction, caspase 3 inactivates the flippases and activates the scramblase XKR8 (Suzuki et al., 2013) that moves the PS to the plasma membrane’s outer leaflet. PS is recognized by several phagocytic receptors or bridging molecules that facilitate the engulfment of the apoptotic cell. While *in vitro* apoptosis often turns into secondary necrosis, *in vivo* dying cells are very quickly cleared by macrophages (Nagata, 2018), usually before the appearance of some of the classical features of apoptosis such as nuclear condensation and blebbing (Dzhagalov et al., 2013).

Fas-induced cell death plays an essential role in the immune system. Cytotoxic CD8^+^ T lymphocytes and NK cells use it to destroy target cells, and effector T cells are eliminated through Fas ligation during chronic infection (Strasser et al., 2009). However, its role in T cell development is controversial. Initial studies suggested that Fas might be necessary to eliminate autoreactive developing T cells in the thymus (negative selection), particularly at high antigen doses (Castro et al., 1996; Kishimoto and Sprent, 1997; Kishimoto et al., 1998). However, later work demonstrated that the absence of Fas, or FADD, or caspase 8 in T cells does not lead to defects in negative selection (Newton et al., 1998; Salmena et al., 2003; Hao et al., 2004). Thus, at present, the role of Fas in central tolerance is doubtful.

Understanding the regulation of apoptosis is of enormous interest because of its potential therapeutic implications ranging from cancer to autoimmune diseases. The main molecular players in the process have been identified, and *in vitro* apoptosis has been extensively researched and modeled (Spencer and Sorger, 2011). These studies have revealed that different cells, even within a clonal population, undergo outer mitochondrial membrane permeabilization and caspase activation at different times (Goldstein et al., 2000). Despite this progress, it is still unclear what is the apoptosis dynamics *in vivo*. Recently, we described the timeline for thymocyte cell death during negative selection that uses the intrinsic pathway of apoptosis *in situ*, in a tissue explant. We defined the timing of several key apoptosis events such as caspase 3 activation, PS externalization, loss of plasma membrane integrity, and cell loss due to efferocytosis. However, such information is lacking for apoptosis triggered through the extrinsic pathway. Although the kinetics of cell death due to Fas ligation have been described *in vitro* (Ogasawara et al., 1995), and computationally modeled (Hua et al., 2005; Fricker et al., 2010), there is still uncertainty how the tissue environment, specifically the presence of efferocytosis and pro-survival factors such as cytokines can modify the progression of apoptosis proceeding through the extrinsic pathway. A major problem for *in vivo* study of Fas-induced cell death is the broad expression of Fas that leads to the death of experimental animals within hours of injection of stimulating antibodies (Ogasawara et al., 1993) or recombinant FasL (Huang et al., 1999).

Here, we overcame the problem of mortality to study apoptosis induced by Fas ligation *in vivo* using tissue explants that maintain the 3D structure of the thymus and contain macrophages and survival factors. With this system, we determined the timeline of cell death in a cohort of thymocytes receiving simultaneous Fas ligation *in situ*. The timing of caspase 3 activation, PS exposure, and cell membrane permeabilization was similar to intrinsic pathway-induced apoptosis, and just like observed in our previous work (Dzhagalov et al., 2013) and *in vitro* (Albeck et al., 2008) was asynchronous at a single-cell level. Cell loss due to efferocytosis was first detectable 2 h after Fas ligation, and by 8 h >80% of all cells were cleared. Caspase 3 activation and PS exposure were essential for the progression of apoptosis and efferocytosis. Using this model, we also re-examined whether Fas is essential for negative selection to a ubiquitous antigen. In agreement with previous studies (Villunger et al., 2004), we found that this pathway of apoptosis is dispensable for eliminating autoreactive cells in the thymus.

## Materials and Methods

### Mice

C57BL/6Narl mouse was purchased from the National Laboratory Animal Center, NARLabs, Taipei, Taiwan, an AAALAC-accredited facility. OT1 mice were bred out from OT1 UBC-tdTomato (C57BL/6-Tg(TcrαTcrβ)1100Mjb Tg(UBC-tdTomato)1Narl/Narl) mice purchased from the National Laboratory Animal Center, NARLabs, Taipei, Taiwan. *Lpr* mice (B6.MRL-Fas^*lpr*^/J, stock #000482) and *gld* mice (B6Smn.C3-Fasl^*gld*^/J, stock #001021) were purchased from the Jackson Laboratory (Bar Harbor, Maine, United States) (Andrews et al., 1978; Roths et al., 1984). All mice were housed in the animal facility of National Yang-Ming University and used between 6 and 12 weeks of age. All animal experiments were approved by the Institutional Animal Care and Use Committee (IACUC) of National Yang-Ming University.

### Cell Isolation and Labeling

Thymuses from C57BL/6, *lpr*, or OT1 mice were harvested, cleaned of connective tissue, mechanically dissociated with a syringe plunger in 6 cm dishes (Alpha, Taiwan) in PBS, and filtered through 70 μm Nylon mesh (Small Parts). For experiments involving overlaying labeled cells on thymic slices, 10^7^ cells were transferred in a 15 mL conical tube (Jet Biofil), washed with PBS, and resuspended in PBS containing 2 μM Cell Proliferation Dye eFluor450 (ThermoFisher) or 1 μM Cell Proliferation Dye eFluor670 (ThermoFisher). The cell suspension was incubated at 37°C for 15 min, washed with complete DMEM (cDMEM) consisting of high glucose DMEM, 10% fetal bovine serum, 1% L-Glutamine, 1% Penicillin/Streptomycin, and 0.1% 2-mercaptoethanol (all from Gibco) and resuspended in cDMEM at a density of 5 × 10^5^/10 μL. The WT cells labeled with eFluor670 and the *lpr* cells labeled with eFluor450 were mixed at a 1:1 ratio and used for overlaying *lpr* thymic slices for experiments involving sFasL stimulation. Alternatively, WT cells labeled with eFluor670 and OT1 cells labeled with eFluor450 were mixed and used for overlaying thymic slices for negative selection experiments.

### Thymic Slice Preparation and Treatment

Thymic slices were prepared essentially as described (Zhou et al., 2020). Briefly, the thymuses of *lpr*, or *gld*, or C57BL/6 mice were dissected, the two lobes were separated and carefully cleaned out of connective tissue. Individual lobes were embedded in 4% low-melting-point agarose (GTG-NuSieve, Lonza) in HBSS (Gibco). The resulting block was trimmed of excess agarose, glued onto the stage of Vibratome 1000S (Leica), submerged in ice-cold PBS, and cut into 400 μm thick slices. The slices were put on 0.4 μm Cell Culture Inserts (Falcon) in a 6-well tissue culture plate (Falcon) containing 1 mL of cDMEM under the inserts. Each slice was carefully overlaid with 10 μL (∼5 × 10^5^) of a mixture of eFluor450-labeled and eFluor670-labeled thymocytes for 2 h in a 5% CO_2_ incubator at 37°C. After the incubation, each slice was gently washed with cDMEM to remove cells that have failed to penetrate inside it. sFasL (Enzo Life Sciences) was added to the top of the thymic slices in a volume of ∼10 μL at a concentration of 100 ng/mL determined to be optimal for inducing cell death in a slice in pilot experiments. In some cases, 10 μM zDEVD-fmk (APExBIO) was added to sFasL or overlaid on the thymic slices by itself to block caspase 3/7. For blocking PS recognition, the labeled cells were resuspended in cDMEM containing an equal volume of 200 μg/mL purified Annexin V (BioLegend) mixed with 0.2 volumes of 5X Annexin V-binding buffer and overlaid on the thymic slices. After 2 h, the cells were washed out, and sFasL or vehicle was added to 100 ng/mL final concentration in a buffer containing cDMEM and an equal volume of 200 μg/mL purified Annexin V in its binding buffer. At different times, the individual slices were mechanically dissociated with a syringe plunger in FACS buffer [PBS + 0.5% BSA (HM Biological) + 1 mM EDTA (Merck) + 0.1% NaN_3_ (Sigma)] in a 6 cm dish (Alpha, Taiwan) and filtered into FACS tubes (Falcon) for further analysis by flow cytometry.

For induction of negative selection, C57BL/6 thymocytes labeled with eFluor670 and OT1 thymocytes labeled with eFluor450 were mixed at 1:1 ratio and overlaid on C57BL/6 or *gld* slices for 2 h. After the incubation, each slice was gently washed with cDMEM to remove cells that have failed to penetrate inside it. Ova_257__–__264_ peptide (Anaspec) was added to the top of the thymic slices in a volume of ∼10 μL at a concentration of 10 ng/mL. After 12 h, the slices were mechanically dissociated and analyzed by flow cytometry.

### Flow Cytometry

Single-cell suspensions (0.5–2 × 10^6^ cells) from thymuses or thymic slices were blocked with 100 μL supernatant from 2.4G2 hybridoma (a kind gift from Dr. Fang Liao, Academia Sinica, Taipei, Taiwan) and stained with fluorochrome- or biotin-labeled antibodies for 20 min on ice in 100 μL FACS buffer. The following antibodies were used: anti-mouse CD4-FITC (clone GK1.5), anti-mouse CD8α-APC/Fire750 (clone 53–6.7), anti-mouse NK1.1-PE/cy7 (clone PK136), anti-mouse TCRβ-APC (clone H57-597), anti-mouse CD45-PerCP/Cy5.5 (clone 30-F11), anti-mouse CD326 (EpCAM)-APC/Cy7 (clone G8.8), anti-mouse Podoplanin (gp38)-PE/Cy7 (clone 8.1.1), anti-mouse F4/80-BV421 (clone BM8), anti-mouse CD11c-PE/cy7 (clone N418), anti-mouse I-A/I-E (clone M5/114.15.2), CD178 (FasL)-biotin (clone MFL3), and Armenian hamster IgG-biotin (clone HTK888) from BioLegend; Siglec F-AlexaFluor647 (clone E50-2440) from BD Biosciences; CD95 (Fas)-biotin (clone REA453) and recombinant human IgG1-biotin (clone REA293) from Miltenyi Biotech. After the staining, the cells were washed with FACS buffer, and if necessary, incubated for 20 more min on ice with Streptavidin-PE (BioLegend). The cells were then washed again in FACS buffer and resuspended in 300 μL FACS buffer containing 3 mM DAPI (ThermoFisher) to exclude dead cells. The data were acquired on LSR Fortessa (BD Biosciences) flow cytometry running Diva 8 software and analyzed with FlowJo 10.6.2 (BD Biosciences).

For Annexin V staining, after the surface staining, the cells were washed with phenol red-free DMEM (prfDMEM) (Corning) and stained with Annexin V-FITC (BioLegend) used at 1:500 dilution in prfDMEM for 15 min at room temperature in the dark. The cells were washed with prfDMEM and were resuspended in 300 μL prfDMEM containing 3 mM DAPI.

For active caspase 3 staining, after the surface marker staining, the cells were washed with PBS (or prfDMEM if Annexin V was used) and stained with Zombie Aqua (BioLegend) at 1:500 dilution in 500 μL PBS or prfDMEM for 30 min in ice. The cells were washed with FACS buffer or prfDMEM, resuspended in 200 μL PBS, and fixed with 200 μL 4% paraformaldehyde (EMS) in PBS for 20 min in ice. After washing with FACS buffer, the cells were permeabilized with 200 μL 0.1% Saponin (Sigma) in FACS buffer for 20 min in ice. All subsequent washes and incubations were done with 0.1% Saponin (Sigma) in FACS buffer. The cells were again blocked with 10% 24G2 supernatant in 0.1% Saponin (Sigma) in FACS buffer for 10 min in ice and stained with purified anti-active caspase 3 antibody (Cell Signaling Technologies) at 1:400 for 40 min in ice. After 2 washing steps, the staining was revealed with goat anti-rabbit-PE antibody (Jackson Immunoresearch) used at 1:200 dilution for 20 min in ice. After washing, the cells were resuspended in 300 μL 0.1% Saponin (Sigma) in FACS buffer and analyzed by flow cytometry.

### Cell Loss Calculation

To find out the proportion of WT thymocytes lost to efferocytosis, we determined the number of WT and overlaid *lpr* cells among single cells in the lymphocyte gate (all cells). Then, we calculated the ratio of WT to overlaid *lpr* cells. To compare between different experiments, we normalized the ratios, so that the mean of the samples at time point 0 (no treatment) was set to 1. To determine the proportion of WT cells that are recruited into the apoptotic program, we did the same calculation, but we used the number of WT and overlaid *lpr* cells among active caspase 3- (viable) cells. Cell loss due to negative selection was calculated similarly by dividing the number of OT1 thymocytes by WT and normalizing the ratio so that the no peptide group on WT slices was set to 1.

### Immunofluorescent Imaging

The thymic slices were stained and cleared essentially as described (Ghigo et al., 2013). Briefly, the slices were fixed in 4% paraformaldehyde (EMS) for 1 h at room temperature under gentle agitation, then washed with 0.1 M phosphate buffer for 1 h, followed by a wash with 0.1 M TRIS buffer. The slices were blocked with IHC buffer [0.5% bovine serum albumin (Biotium) + 2% Triton X-100 (Sigma) in 0.1M TRIS buffer] for 20 min at room temperature. The primary anti-MerTK purified rat antibody (ThermoFisher) was added in IHC buffer at 1:400 dilution and incubated overnight at 4°C. The slices were washed twice with 0.1M TRIS buffer for 1 h at room temperature and incubated with anti-rat Cy3 secondary antibody (ThermoFisher) overnight at 4°C. After 2 more washes in 0.1M TRIS buffer, the slices were cleared in Rapiclear 1.47 (SunJin) overnight, mounted in Rapiclear 1.47 on microscope slides, covered with a cover glass, and sealed with nail polish. 3D images (*x* = 640 μm, *y* = 640 μm, *z* = 42–66 μm) were acquired with a LSM700 (Zeiss) confocal microscope and were processed with Imaris 8.0.2 (Bitplane). The scoring of phagocytosed cells was done independently by two researchers, and only cells that were scored by both were counted.

### Statistical Analysis

Prism 6.0 (GraphPad) was used for creating graphs and statistical analyses. An unpaired two-tailed *t*-test was used when comparing two groups, and one-way ANOVA with Tukey post-test was applied when more than two groups were compared. All data are presented as mean ± SEM. A *p*-value lower than 0.05 was considered statistically significant.

## Results

### Kinetics of Cell Death-Related Events During FasL-Induced Thymocyte Apoptosis

**FIGURE 1:**
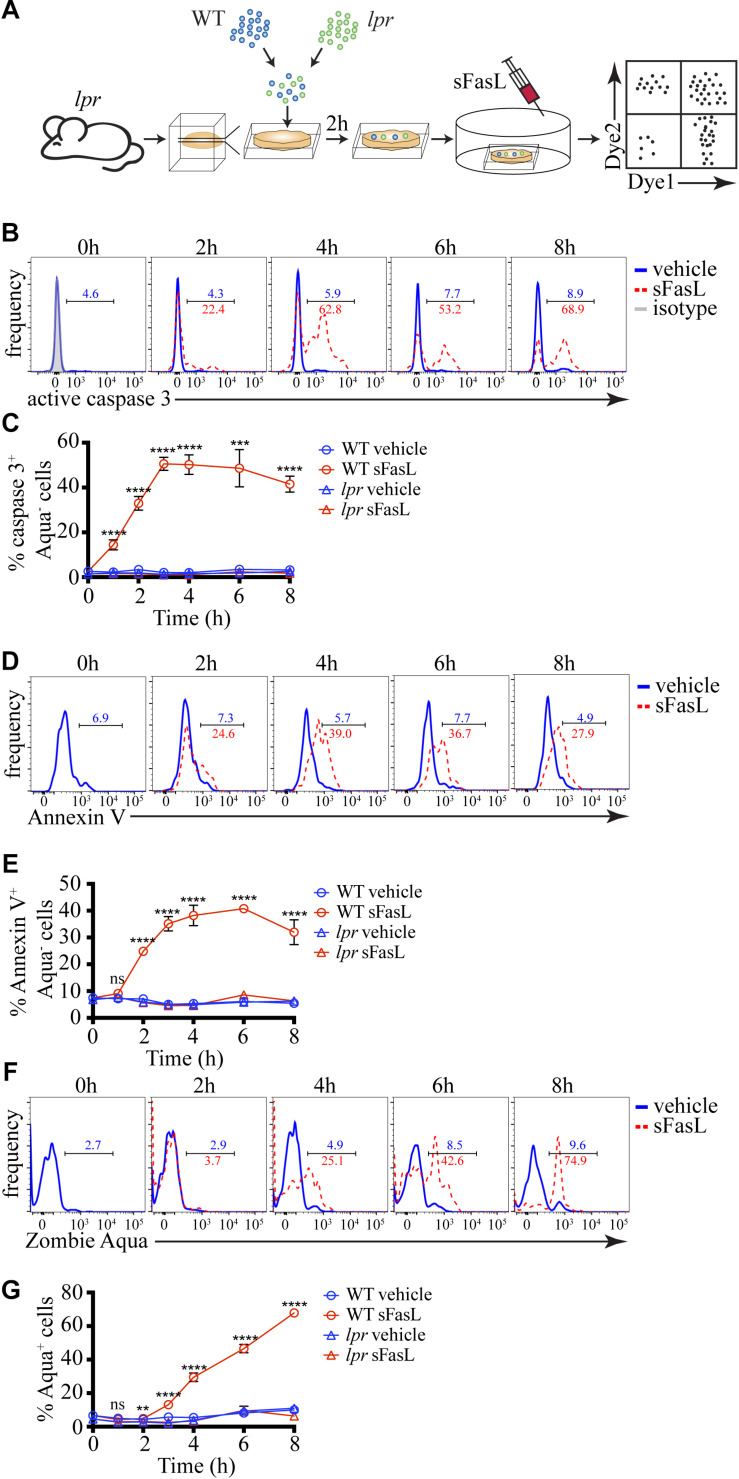
Kinetics of caspase 3 activation, PS exposure, and cell membrane permeabilization of thymocytes treated with sFasL. **(A)** Scheme of the experimental system: differentially labeled C57BL/6 (WT) and *lpr* thymocytes were overlaid on *lpr* thymic slices, and after 2 h, the cells that have failed to penetrate the slices were washed off. 100 ng/mL sFasL was added to the slices, and after different times they were dissociated and analyzed by flow cytometry. **(B)** Flow cytometry plots of caspase 3 activation in Zombie Aqua^–^ WT thymocytes at different times after sFasL addition. The background is established by isotype control (gray). **(C)** Time course of caspase 3 activation among Zombie Aqua^–^ cells in different experimental groups. **(D)** Flow cytometry plots of Annexin V staining of Zombie Aqua^–^ WT thymocytes at different time points after sFasL addition. **(E)** Time course of Annexin V staining among Zombie Aqua^–^ cells in different experimental groups. **(F)** Flow cytometry plots of Zombie Aqua staining of WT thymocytes at different time points after sFasL addition. **(G)** Time course of Zombie Aqua staining among single cells in different experimental groups. The numbers inside the plots are the percentages of positive cells in the vehicle treatment group (blue) and sFasL group (red). The data in **(C,E,G)** are mean ± SEM, and plots in **(B,D,F)** are representative from 3–8 independent experiments with 3 slices each. Statistical significance was determined with one-way ANOVA with Tukey post-test, **p < 0.01, ***p < 0.001, ****p < 0.0001, ns, not significant.

To examine the kinetics of FasL-induced cell death *in situ*, we adapted our previously published experimental system (Figure 1A) for studying negative selection (Dzhagalov et al., 2013). As a death-inducing stimulus, we used SuperFas ligand (sFasL) – an oligomeric form of FasL that can efficiently induce clustering of Fas and initiation of the extrinsic pathway of apoptosis without the use of additional reagents (Shiraishi et al., 2004). To ensure that only a small fraction of cells will undergo programmed cell death, thus avoiding massive apoptosis, we took advantage of *lpr* mice that have a mutation in Fas and are unresponsive to FasL (Watanabe-Fukunaga et al., 1992). We prepared Vibratome-cut 400 μm thick slices from *lpr* thymus and overlaid them with a mixture of differentially labeled WT and *lpr* thymocytes. After 2 h, when many of the overlaid cells have penetrated the thymic slice, we added 100 ng/mL sFasL that was expected to induce apoptosis in WT thymocytes, but not in *lpr* cells. At different times, we dissociated the slices and determined the kinetics of caspase 3 activation, PS exposure, and cell membrane permeabilization. The presence of labeled *lpr* cells controlled for the efficiency of penetration into the slices and non-specific cell death as they were not affected by the sFasL.

Caspase 3 activation is one of the early signs of apoptosis. Within 1 hour of sFasL stimulation, we could detect ∼15% of WT thymocytes activating caspase 3 (Figures 1B,C). The peak of caspase 3 activation was reached at 3 h when around half of all WT thymocytes were positive and did not change much until our last time point −8 h. For comparison, WT thymocytes not treated with sFasL or *lpr* thymocytes with or without sFasL did not show caspase 3 activation above background, confirming our experimental system’s high sensitivity. PS exposure on the cell membrane that can be detected by Annexin V is also considered an early sign of apoptosis but is dependent on caspase 3 activation (Segawa et al., 2014). Indeed, the kinetics of PS externalization was slightly slower than caspase 3 activation: at 1 h after sFasL addition, there was no increase in Annexin V^+^ WT thymocytes, but by 2 h ∼25% of these cells were positive (Figures 1D,E). The peak was reached between 3 and 6 h after sFasL addition. Again our 3 control groups showed no increase in Annexin V^+^ cells above background. Finally, we also measured the kinetics of loss of cell membrane integrity, a late event in the progression of apoptosis, with the dye Zombie Aqua. We could first detect Zombie Aqua^+^ cells at 3 h after sFasL addition, and the proportion of these cells kept increasing until the final time point when ∼75% of all WT thymocytes lost their cell membrane integrity (Figures 1F,G). We speculate that the high level of secondary necrosis at later time points represents an artifact of the isolation procedure. Cell undergoing apoptosis, with activated caspase 3, could be more sensitive to the mechanical disruption we use to liberate them from the slices and their membranes could break down in the process. Thus, we conclude that in our experimental system, caspase 3 activation occurs within 1 h of sFasL addition. It is followed shortly by PS externalization that is evident within 2 h. Cell membrane permeabilization starts to occur after 3–4 h. This timeline is similar to negative selection that uses the intrinsic pathway of apoptosis (Dzhagalov et al., 2013). Moreover, just like in negative selection (Dzhagalov et al., 2013) or *in vitro* (Albeck et al., 2008), although the death-inducing stimulus is applied simultaneously to all cells, apoptosis proceeds asynchronously.

### Cell Loss Occurs Rapidly Following sFasL Stimulation

**FIGURE 2:**
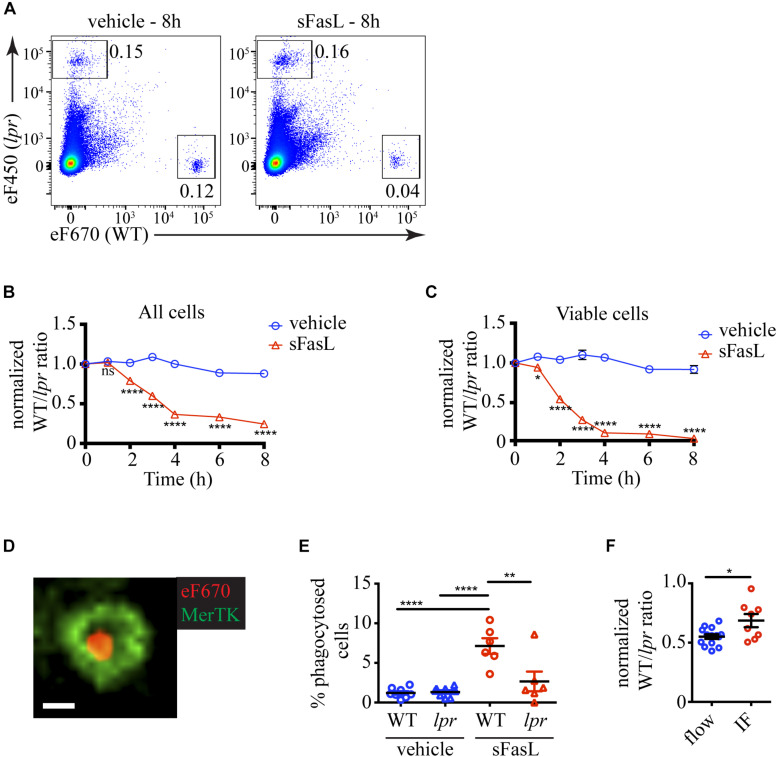
Kinetics of cell loss of thymocytes treated with sFasL. **(A)** Flow cytometry plots showing the proportions of overlaid WT and *lpr* thymocytes in slices treated with sFasL and control (vehicle) at the final time point (8 h) of the experiment. The plots are representative of 9–12 slices from 3–4 independent experiments. The numbers inside the plots are the percentages of the cells in the respective gate. **(B)** Time course of cell loss among single cells in the lymphocyte gate (all cells). **(C)** Time course of cell loss among active caspase 3^–^ (viable) cells. **(D)** Example confocal image of a eFluor 670-labeled WT thymocyte (eF670) in red engulfed by a MerTK^+^ macrophage (green). The scale bar is 5 μm. The image is representative of more than 50 examples. **(E)** Comparison of the percentages of engulfed cells of *lpr* or WT genotype in slices treated for 4 h with 100 ng/mL sFasL or vehicle. **(F)** Comparison of the cell loss measured by flow cytometry (flow) and immunofluorescent imaging (IF) at 4 h. The data are normalized to vehicle control in each experiment that is set to 1. The data in **(B,C)** are mean ± SEM from 3–8 independent experiments with 3 slices each. The data in **(E,F)** are mean ± SEM from 3 independent experiments with 2–3 slices each. For the imaging, two 3D volumes were recorded in each slice. Each symbol is an individual slice (for flow) or individual 3D imaging volume (IF). Statistical significance was determined with unpaired two-tailed *t*-test for **(B,C,F)**, or one-way ANOVA with Tukey post-test for **(E)**, *p < 0.05, **p < 0.01, ****p < 0.0001, ns, not significant.

Because apoptotic cells are rapidly removed by efferocytosis, we reasoned that the proportion of apoptotic cells at any time would depend on the numbers of (1) newly recruited into the apoptotic program cells, (2) existing apoptotic cells, and (3) apoptotic cells removed by efferocytosis. A distinct advantage of our experimental system is that it contains phagocytes and allows us to quantify the natural end-point of apoptosis *in vivo* – efferocytosis. The presence of labeled *lpr* thymocytes that should not respond to sFasL, but will remain sensitive to non-specific cell death-inducing stimuli provides an excellent background internal control (Figure 2A). The change in the WT/*lpr* ratio relative to time point 0 will reflect the extent of WT cell loss by efferocytosis. The ratio between all WT and *lpr* thymocytes started decreasing 2 h after sFasL addition and reached ∼0.3 after 4 h, indicating that ∼70% of all WT cells have been lost by that time point (Figure 2B). We then focused our attention on the cells showing no signs of apoptosis and found out that within the first hour after sFasL addition, the WT/*lpr* ratio started to decline, and within 4 h only ∼10% of WT cells showed no sign of apoptosis (Figure 2C).

To prove that the cell loss we observed was due to efferocytosis, we tried to use flow cytometry, but we did not detect a clear population of macrophages containing labeled cells. Then we turned to imaging. We cleared and stained the thymic slices for MerTK, a well-established marker for macrophages (Gautier et al., 2012). We could, indeed, observe cells engulfed in macrophages (Figure 2D and Supplementary Movie 1). There was a significantly higher percentage of phagocytosed WT cells on sFasL-treated slices than WT cells on untreated slices or *lpr* cells (Figure 2E), confirming that efferocytosis is a major contributor to cell loss. Consistent with this idea, the percentage of cell loss determined by imaging was significantly lower than the cell loss determined by flow cytometry (Figure 2F). The difference was mostly due to phagocytosed cells. However, the imaging experiments also documented significant cell loss, suggesting that many cells have already been degraded and can no longer be detected on the slice. Thus, our results show that sFasL treatment caused the phagocytosis of WT cells that could be measured as cell loss. The cell loss and the overall decline in viability caused by sFasL followed a time course similar to the apoptosis markers we examined in Figure 1.

### sFasL-Induced Thymocytes Cell Death Depends on Caspase 3 Activation *in situ*

**FIGURE 3:**
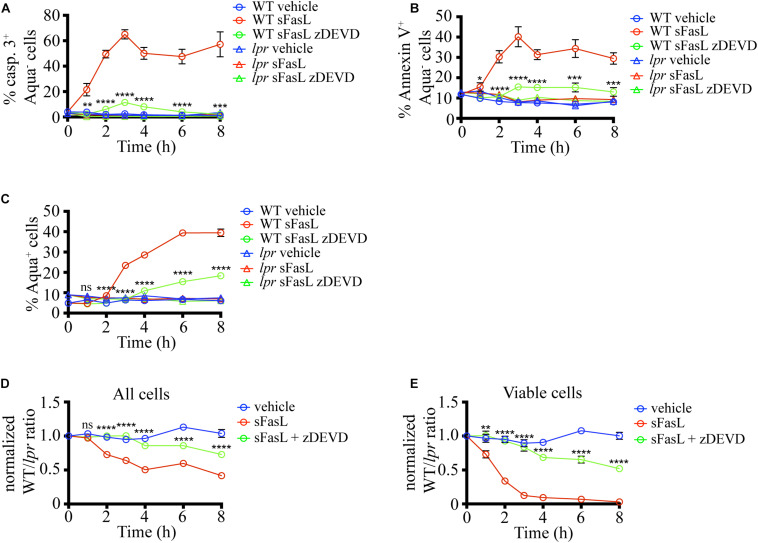
Caspase 3/7 inhibitor (zDEVD) abolishes PS exposure, plasma membrane permeabilization, and cell loss in response to sFasL. **(A)** Time course of caspase 3 activation among Zombie Aqua^–^ cells in different experimental groups. **(B)** Time course of Annexin V staining among Zombie Aqua^–^ cells in different experimental groups. **(C)** Time course of Zombie Aqua staining among different experimental groups. **(D)** Time course of cell loss among single cells in the lymphocyte gate (all cells). **(E)** Time course of cell loss among active caspase 3- (viable) cells. The data in **(A)** are mean ± SEM from 2–5 independent experiments with 3 slices each. The data in **(B–E)** are mean ± SEM from 4–10 independent experiments with 3 slices each. Statistical significance of the difference between WT + sFasL and WT + sFasL + zDEVD groups was determined with unpaired two-tailed *t*-test, *p < 0.05, **p < 0.01, ***p < 0.001, ****p < 0.0001.

Next, we wanted to dissect the critical steps in the apoptosis program induced by sFasL *in situ*. The extrinsic death pathway triggered by Fas ligation activates caspase 3, a crucial executioner of the death pathway, cleaving hundreds of proteins to bring the demise of the cell (Nagata, 2018). To evaluate the importance of caspase 3 for the progression of FasL-induced apoptosis *in situ*, we added the caspase 3/7 inhibitor zDEVD-fmk (zDEVD) together with sFasL to the thymic slices containing labeled WT and *lpr* thymocytes. As expected, zDEVD efficiently suppressed caspase 3 activation in WT cells upon sFasL ligation (Figure 3A). In addition, zDEVD also abolished PS externalization in response to FasL, showing that PS exposure depends on caspase 3/7 (Figure 3B). Finally, zDEVD greatly slowed down the appearance of cells with permeabilized plasma membrane, as these could only be observed 6 h after the addition of the sFasL (Figure 3C). Thus, caspase 3 inhibition suppressed all signs of apoptosis induced by Fas ligation, confirming that it is critical in the progression of this mode of cell death.

We also tested if caspase inhibition can interfere with the cell loss following sFasL treatment. The addition of zDEVD significantly slowed down cell loss (Figure 3D). The first signs of cell loss were detectable 6 h after sFasL addition in the presence of zDEVD, instead of at 2 h. These results suggest that the efferocytosis of cells treated with sFasL depends on caspase 3/7 activation. Very similar data were obtained when the loss of viable cells was compared (Figure 3E). More than 50% of the overlaid cells did not show any signs of apoptosis after 8 h of sFasL treatment in the presence of zDEVD, compared to <5% in its absence. Our treatment’s inability to completely suppress the loss of cell membrane integrity and the cell loss could be due to the degradation of the drug at later time points or the activity of additional members of the caspase family that are not efficiently blocked by zDEVD.

### PS Exposure Is Critical for Cell Loss *in situ*

**FIGURE 4:**
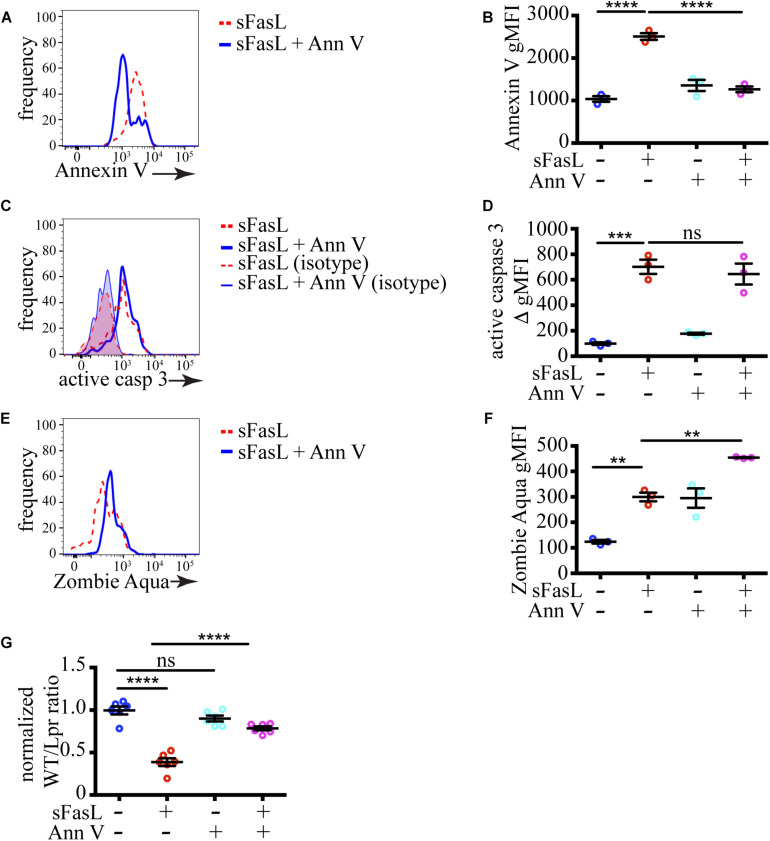
Masking of PS abolishes cell loss. **(A)** Histograms showing PS exposure on WT thymocytes treated with sFasL with or without 200 μg/mL unlabeled Annexin V for 12 h. **(B)** Comparison of geometric mean fluorescent intensity (gMFI) of Annexin V staining on WT cells after treatment with sFasL and unlabeled Annexin V for 12 h. **(C)** Histogram overlays of caspase 3 activation in WT thymocytes treated with sFasL with or without unlabeled Annexin V for 12 h. The background is established with isotype controls (thin lines). **(D)** Comparison of caspase 3 activation in WT cells after treatment with sFasL and unlabeled Annexin V for 12 h. The staining was quantified by subtracting the gMFI of isotype control from the gMFI of active caspase 3 (ΔgMFI). **(E)** Histograms showing Zombie Aqua staining on WT thymocytes treated with sFasL with or without unlabeled Annexin V for 12 h. **(F)** Comparison of the gMFI of Zombie Aqua staining in WT cells after treatment with sFasL and unlabeled Annexin V for 12 h. **(G)** Comparison of cell loss among single cells in the lymphocyte gate after treatment with sFasL and unlabeled Annexin V for 12 h. The data in **(A,C,E)** are representative of 4 independent experiments with 3 slices each. The data in **(B,D,F)** are mean ± SEM from a representative experiment out of 4 independent experiments. The data in **(G)** is mean ± SEM compiled from 2 independent experiments with 3 slices each. Each dot is an individual slice. Statistical significance was determined with one-way ANOVA with Tukey post-test, **p < 0.01, ***p < 0.001, ****p < 0.0001, ns, not significant.

To test if PS exposure plays a role in the progression of thymocytes cell death induced by sFasL, we blocked PS recognition on apoptotic cells by incubation with excess unlabeled Annexin V. Annexin V binds PS and prevents its interaction with PS receptors on phagocytes (Kurd et al., 2019). As we expected, the amount of PS that could be detected after pre-incubation with 200 μg/mL unlabeled Annexin V was significantly reduced on sFasL treated WT thymocytes (Figures 4A,B), confirming that unlabeled Annexin V can mask PS. The extent of caspase 3 activation was unchanged by Annexin V addition (Figures 4C,D), consistent with our data in Figure 3 that caspase 3 is upstream of PS externalization. However, the extent of labeling with Zombie Aqua was increased by Annexin V treatment (Figures 4E,F), suggesting that cells with compromised membrane integrity were accumulating instead of being engulfed by macrophages. This idea was confirmed when we calculated cell loss. The addition of Annexin V to sFasL rescued the cell loss (Figure 4G), most likely by preventing the efferocytosis. Importantly, Annexin V, without sFasL, had little effect on caspase 3 activation or cell loss. Lower concentration of Annexin V (60 μg/mL) could not efficiently mask surface PS on apoptotic cells, and had no effect on cell loss (Supplementary Figure 1), suggesting that there is a threshold of PS exposure necessary for successful engulfment. Thus, the data from Annexin V blocking experiments show that PS externalization is critical for the timely efferocytosis and that PS is the most important “eat-me” signal displayed by dying cells treated with sFasL.

### Abundant Expression of Fas, but Minimal Expression of FasL on Cells in the Thymus

**FIGURE 5:**
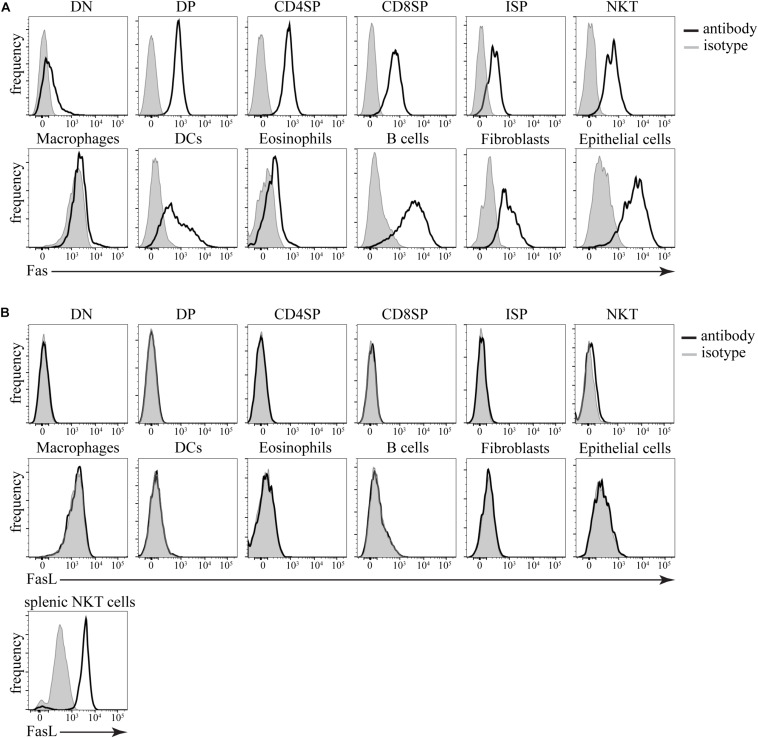
Expression of Fas and FasL in the thymus. **(A)** Histograms showing expression of Fas in different cell types. **(B)** Histograms showing expression of FasL in different cell types. Isotype control (gray) defines background. The data are representative of 4 independent experiments. DN – CD4^–^CD8^–^ double-negative thymocytes, DP – CD4^+^CD8^+^ double-positive thymocytes, CD4SP – CD4^+^CD8^–^ single-positive thymocytes, CD8SP – CD4^–^CD8^+^TCRβ^+^ single-positive thymocytes, ISP – CD4^–^CD8^+^TCRβ^–^ immature single-positive thymocytes, NKT – NK1.1^+^TCRβ^+^ natural killer T cells, Macrophages – F4/80^+^, dendritic cells (DCs) – CD11c^+^MHC2^+^, Eosinophils – Siglec F^+^, B cells – CD11c^–^MHC2^+^, fibroblasts – CD45^–^gp38^+^, epithelial cells – CD45^–^EpCAM^+^. As a positive control, splenic NKT cells (TCRβ^+^NK1.1^+^) from *lpr* mice were used.

Armed with the detailed knowledge of the kinetics of Fas-induced cell death in this experimental system, we wanted to test if this mode of dying has physiological relevance in the thymus. First, we tested the expression of Fas and FasL on various cells in the thymus by flow cytometry. We found that Fas was expressed on virtually every cell type that we tested (Figure 5A). This included all subpopulations of thymocytes, such as CD4^–^CD8^–^ double-negative, CD4^+^CD8^+^ double-positive, CD4^+^CD8^–^ CD4 single-positive, CD4^–^CD8^+^TCRβ^+^ CD8 single-positive, CD4^–^CD8^+^TCRβ^–^ immature single-positive cells, and NK1.1^+^ TCRβ^+^ natural killer T (NKT) cells. All major supporting cell types such as macrophages, dendritic cells, eosinophils, B cells, thymic epithelial cells, and thymic fibroblasts were also positive for Fas expression. However, when we examined FasL expression, we found that the only cells that expressed any FasL were the NKT cells (Figure 5B). Although there are reports of FasL detection on thymocytes by flow cytometry (Boursalian and Fink, 2003), our results are entirely consistent with the data from IMMGEN (Supplementary Figure 2) that show that only NKT cells in the thymus express FasL mRNA.

### Absence of FasL Does Not Affect Negative Selection *in situ*

**FIGURE 6:**
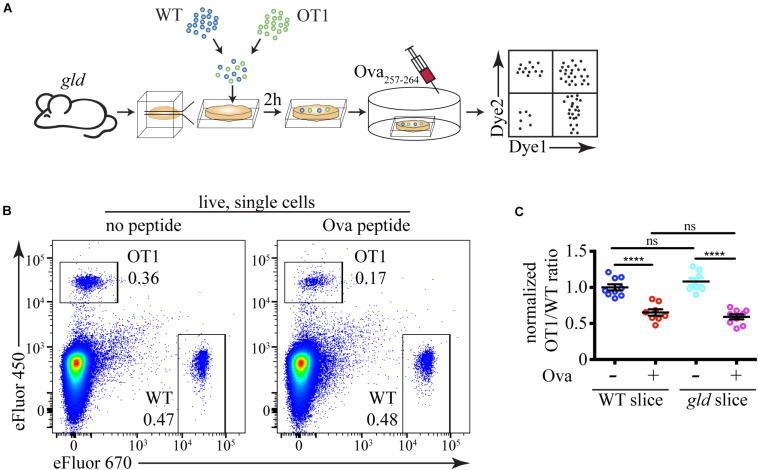
FasL does not have a critical role during negative selection. **(A)** Scheme of the experimental system: differentially labeled C57BL/6 (WT) and OT1 thymocytes were overlaid on *gld* thymic slices, and after 2 h, the cells that have failed to penetrate the slices were washed off. The negative selection was induced by the addition of 10 ng/mL Ova_257__–__264_ peptide (Ova) to the slices, and after 12 h, the slices were dissociated and analyzed by flow cytometry. **(B)** Flow cytometry plots showing the proportions of WT and OT1 thymocytes in slices from *gld* mice treated with or without Ova for 12 h. The plots are representative of 9 slices from 3 independent experiments. The numbers inside the plots are the percentages of the cells in the respective gate. **(C)** Comparison of cell loss among OT1 single cells in the lymphocyte gate after treatment with Ova on WT slices in the presence of FasL and on *gld* slices in the absence of FasL. The data in **(C)** is mean ± SEM compiled from 3 independent experiments with 3 slices each. Each dot is an individual slice. Statistical significance was determined with one-way ANOVA with Tukey post-test, ****p < 0.0001, ns, not significant.

Finally, we used our sensitive experimental system to address the issue if Fas ligation plays an important role in negative selection of autoreactive thymocytes. We prepared thymic slices from *gld* mice that lack functional FasL and overlaid them with differentially labeled WT and OT1 thymocytes (Figure 6A). OT1 thymocytes express a transgenic T cell receptor that recognizes a peptide derived from chicken ovalbumin (Ova_257__–__264_) presented by H2-K^*b*^ (Hogquist et al., 1994). These cells undergo negative selection upon encounter with the Ova_257__–__264_ peptide. If FasL is necessary for the clonal deletion of autoreactive thymocytes, we expect that the loss of OT1 cells will be lower on *gld* slices than on WT slices. However, we found that the cell loss following Ova_257__–__264_ addition to WT and *gld* slices was identical (Figures 6B,C). Thus, our data rule out a critical role for FasL in negative selection.

## Discussion

Studying the kinetics of Fas-induced cell death *in vivo* has been challenging because of the dangers of systemic Fas activation. Here, we tried to address this gap in our knowledge by using a novel tissue explant model. Our results show that within 1 h of Fas ligation, caspase 3 gets activated in thymocytes *in situ*, followed by PS exposure between 1 and 2 h, and loss of membrane integrity starting after 2 h. Cell loss due to efferocytosis coincided with PS exposure and was evident after 1 h of Fas ligation. Death was asynchronous with some cells’ demise occurring as early as 1 h after stimulation, while others survived for at least 8 h. Apoptosis and efferocytosis were dependent on caspase 3 activation, while cell loss was greatly reduced by masking PS on the surface of dying cells. Using this sensitive system, we also determined that Fas ligation does not play a measurable role in a negative selection model. Altogether, this work enhances our knowledge of apoptosis progression in response to death receptor triggering in the tissue environment and confirms that Fas is not a major player in negative selection.

Our results allow us to compare the kinetics of thymocytes cell death between the extrinsic pathway and the intrinsic pathway (Dzhagalov et al., 2013). The sequence of events and their timing are altogether similar. However, Fas ligation resulted in faster caspase 3 activation (within an hour) compared to negative selection (between 1 and 2 h). The faster speed of the extrinsic pathway is not surprising, having in mind the fewer intermediaries between the cell surface receptor and caspase 3 activation. PS exposure occurred at roughly the same time in both models, between 1 and 2 h, but the magnitude at 2 h was greater for Fas ligation. The higher proportion of PS^+^ cells translated into faster cell loss and efferocytosis in this model, starting at 2 h, compared to 3 h in the negative selection model. Moreover, cell death by Fas ligation demonstrated the same asynchronicity as TCR triggering in autoreactive cells did in our previous work (Dzhagalov et al., 2013). Thus, our current data confirm *in situ* the general validity of the “fractional killing” idea (Roux et al., 2015) that simultaneous encounter of a death-inducing stimuli does not kill all of the cells in a population due to stochastic variation in the levels of apoptosis regulators (Spencer et al., 2009). It is important to note that this kinetics comparison is more applicable to pharmacological interventions such as drug delivery than to physiological situations, in which we have very little knowledge about actual concentrations of the ligands.

In this study, we visualized and quantified the efferocytosis of dying cells, highlighting the unique advantages of having an intact tissue microenvironment to study cell death. sFasL treatment caused a significant increase in the engulfment of WT cells by MerTK^+^ macrophages. However, it is also possible that other cells not expressing MerTK, such as dendritic cells, could phagocytose apoptotic thymocytes. Thus, our measurement of the percentage of engulfed cells is most likely an underestimation. Nevertheless, it is also evident that even if we consider all detectable cells, sFasL treatment causes a reduction in the number of WT cells. The rapid degradation of phagocytosed cells could explain the absence of these cells.

A caspase 3/7 inhibitor almost entirely blocked Fas-induced thymocyte death, highlighting the central role of caspases 3 and 7 in apoptosis proceeding through the extrinsic pathway (Lakhani, 2006; McComb et al., 2019). However, starting at 6 h after Fas ligation, we could detect an increase in cell membrane permeabilization and cell loss in the presence of zDEVD. This increase in cell death could be due to the degradation of zDEVD and the decrease in its effective concentration, or the action of other caspases that might be able to substitute for caspases 3/7.

While the importance of caspase 3/7 for apoptosis is well-appreciated, the relative contribution of PS versus other “eat-me” signals has not been explored in depth. PS is the most well-known engulfment signal for efferocytosis. It is recognized by several cell surface receptors such as Tim4, BAI, and Stab2, and bridging molecules such as Protein S, Gas6, MFGE8, and C1q (Fond and Ravichandran, 2016). However, many other molecules have been proposed to serve as “eat-me” signals, such as calreticulin, Annexin I, ICAM3 (Moffatt et al., 1999; Arur et al., 2003; Gardai et al., 2005). Using the Annexin V masking of PS, we were able to dissect the relative contribution of PS vs. other “eat-me” signals. We found that PS is by far the most important “eat-me” signal in our experimental system, and interfering with it almost completely abolished cell loss and efferocytosis. The result is similar to our findings in the negative selection of thymocytes model that proceeds through the intrinsic pathway (Kurd et al., 2019), and suggests that efferocytosis of thymocytes is mostly mediated by PS recognition.

The general agreement in the literature is that Fas is broadly expressed on most cell types in the thymus (Nishimura et al., 1995; Ogasawara et al., 1995). Our data confirm that all populations that we examined would be sensitive to FasL-induced killing. However, it is not so clear what cells, if any, express FasL. One study reported expression restricted to the stromal compartment in the thymus (French et al., 1997), while others claim broad expression, including on most thymocytes subpopulations (Boursalian and Fink, 2003). Our results show that FasL surface expression in the thymus is restricted to NKT cells. Although contradicting some of the earlier reports, these data are in agreement with the IMMGEN database^1^. Minimal FasL expression in the thymus is also easier to reconcile with the ubiquitous Fas presence and the apparent lack of involvement for Fas-FasL interaction in negative selection in the thymus (Singer and Abbas, 1994; Villunger et al., 2004).

Although there have been several reports that Fas is involved in the negative selection of autoreactive thymocytes, particularly at high antigen doses (Castro et al., 1996; Kishimoto and Sprent, 1997; Kishimoto et al., 1998), more recent studies have excluded a role for Fas and its signaling pathway components in central tolerance (Newton et al., 1998; Salmena et al., 2003; Villunger et al., 2004). The consensus nowadays is that negative selection uses the intrinsic pathway of apoptosis, as evidenced by autoreactive cells’ failure to die in Bim^–/–^ mice (Bouillet et al., 2002; Villunger et al., 2004; Gray et al., 2012). Our results confirm this notion with two key findings: the minimal expression range of FasL in the thymus that is insufficient for eliminating the numerous autoreactive cells, and the lack of effect of the absence of FasL on the cell loss due to negative selection.

In summary, our work provides a detailed kinetic analysis of the progression of cell death initiated by Fas ligation *in situ* and identifies the dependence of this apoptosis program on caspase 3/7 activation and PS exposure. We also demonstrate that although almost all thymocytes are competent to undergo Fas-induced cell death, this mode of apoptosis is not involved in the negative selection of autoreactive thymocytes.

## Data Availability Statement

All datasets presented in this study are included in the article/Supplementary Material.

## Ethics Statement

The animal study was reviewed and approved by Institutional Animal Care and Use Committee (IACUC) of National Yang-Ming University.

## Author Contributions

C-FC, H-KF, and ID designed and performed the experiments, analyzed the data, and interpreted the results. K-HS provided *lpr* mice. C-LH designed the experiments and interpreted the data. ID supervised the study and wrote the manuscript with input from the co-authors. All authors contributed to the article and approved the submitted version.

## Conflict of Interest

The authors declare that the research was conducted in the absence of any commercial or financial relationships that could be construed as a potential conflict of interest.

## Footnotes

1 www.immgen.org

## Abbreviations

NKT cells: natural killer T cells
PS: phosphatidylserine
sFasL: SuperFas ligand
zDEVD: zDEVD-fmk.
