# Supplementary material for: Examination of Fas-Induced Apoptosis of Murine Thymocytes in Thymic Tissue Slices Reveals That Fas Is Dispensable for Negative Selection
Source: Front Cell Dev Biol. 2020 Oct 21;8:586807. doi: 10.3389/fcell.2020.586807 (PMC7609743; doi:10.3389/fcell.2020.586807)
Supplement: Supplementary file 2 [file Data_Sheet_1.PDF]

Supplementary figure 1: Expression of FasL in cells in the thymus and other lymphoid organs. Mean expression data from IMMGEN Microarray database for FasL is plotted. The data for DN thymocytes are the average of all DN populations (n=12). The data for DP are the average of 12 samples. The data for CD4SP and CD8SP are the average of all subpopulations (n=9). For the remaining samples, the data are the averages of 3 samples. As the IMMGEN microarray database does not have data on thymic macrophages, B cells, and eosinophils, we included FasL expression in splenic macrophages, splenic follicular B cells, and blood eosinophils.

Supplementary figure 2: Low dose of Annexin V cannot prevent cell loss of WT cells in slices treated with sFasL. (A) Time course of normalized cells of WT thymocytes on thymic slices treated with 100 ng/mL sFasL with or without 60 µg/mL unlabeled Annexin V. The data is mean±SEM from 2 independent experiments with 3 slices each (B) Comparison of Annexin V levels on WT and *lpr* cells in slices treated with 100 ng/mL sFasL with or without 60 µg/mL Annexin V at the 4 h time point. The data is mean±SEM form a representative experiment with 3 slices. Each symbol is an individual thymic slice. Statistical significance in (B) was determined by unpaired t-test, ***p<0.001, ns – not significant.
