# Supplementary material for: Examination of Fas-Induced Apoptosis of Murine Thymocytes in Thymic Tissue Slices Reveals That Fas Is Dispensable for Negative Selection
Source: Front Cell Dev Biol. 2020 Oct 21;8:586807. doi: 10.3389/fcell.2020.586807 (PMC7609743; doi:10.3389/fcell.2020.586807)
Supplement: Supplementary file 3 [file Data_Sheet_2.PDF]

Supplementary movie 1: A 360° rotation of a 3D imaging volume from an *lpr* thymic slice overlaid with eFluor 670 labeled WT cells (red) and eFluor 450 labeled *lpr* cells (blue) treated with sFasL for 4 h. The slice was stained with MerTK antibody to reveal macrophages (green). The arrowheads point to three examples of WT cells inside $MerTK^+$ cells. The scale bar is 10 μm.
