# Supplementary figures and images for: Examination of Fas-Induced Apoptosis of Murine Thymocytes in Thymic Tissue Slices Reveals That Fas Is Dispensable for Negative Selection
Source: Front Cell Dev Biol. 2020 Oct 21;8:586807. doi: 10.3389/fcell.2020.586807 (PMC7609743; doi:10.3389/fcell.2020.586807)

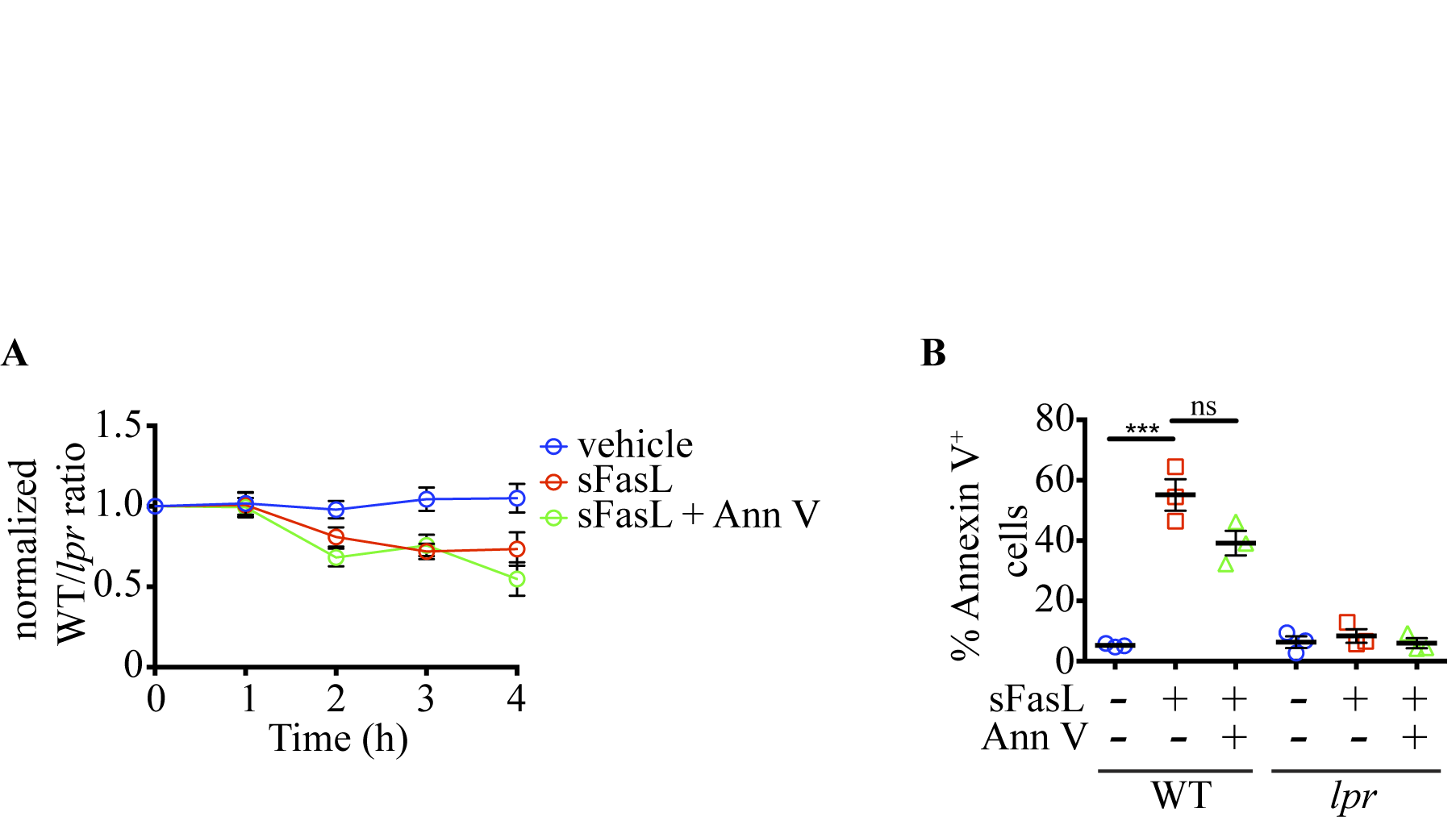

Supplement: Supplementary file 4 [file Image_1.tif]

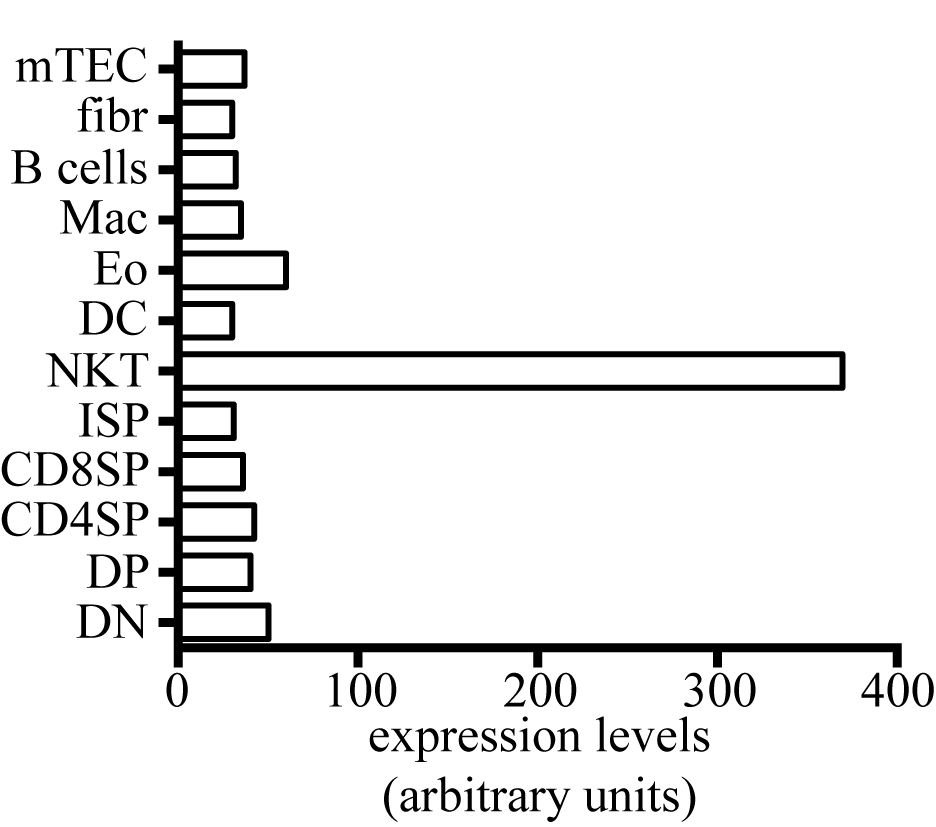

Supplement: Supplementary file 5 [file Image_2.tif]
